# Role of Ultrasonography in the Preoperative Assessment of Impalpable Testes: A Single Center Experience

**DOI:** 10.5402/2012/560216

**Published:** 2012-03-22

**Authors:** Tariq O. Abbas, Noora Al-Shahwani, Ahmed Hayati, Abdul Hady Samaha, Ibrahim E. Bassiouny, Mansour Ali

**Affiliations:** ^1^Pediatric Surgery Department, Hamad General Hospital, Doha 3050, Qatar; ^2^Urology Department, Hamad General Hospital, Doha 3050, Qatar

## Abstract

*Background*. Abdominoscrotal sonogram is often used in boys with a nonpalpable testis to determine the presence of the testis. We describe our experience with the use of ultrasonography in boys with a nonpalpable testis. *Methods*. We retrospectively reviewed the medical records of boys aged less than 14 years, who underwent preoperative ultrasonography for an impalpable undescended testis (IUDT) between 2006 and 2010 in our institution. The results of sonography and laparoscopy were compared. *Results*. A total of 26 impalpable testes in 22 patients, including 4 with bilateral impalpable testes, were preoperatively assessed by ultrasonography for their localization. Sonography localized only 6 of the 26 (23%) testes, which were laparoscopically explored and followed by orchiopexy. *Conclusion*. Ultrasound is not reliable in the preoperative assessment of patients with impalpable testes.

## 1. Introduction

About 1-2% of boys at age 1 year have an undescended testis (UDT); this disorder is unilateral in about 90% of cases and bilateral in about 10% [1–3]. Almost one-fifth of undescended testes are nonpalpable [4]. Approximately 50% of nonpalpable testes are abdominal, with 45% being atrophic secondary to in utero spermatic cord torsion, and 5% being in the inguinal canal [5–7].

Among the radiologic methods used to try to “localize” the nonpalpable testis preoperatively are retrograde venography, computerized tomography (CT), magnetic resonance imaging (MRI), and sonography [8–12]. Abdominoscrotal sonography appears promising for the evaluation of impalpable testes, as it is noninvasive, with no radiation risk, and does not require sedation or general anesthesia. We describe our experience with ultrasonography in the preoperative diagnosis of impalpable testes.

## 2. Methods

We retrospectively reviewed the medical records of all patients less than 14 years who underwent abdominoscrotal ultrasonography for impalpable undescended testes (IUDT) between 2006 and 2010 at our institution. Data reviewed included patient age at surgery, ultrasound results, and intraoperative laparoscopic findings. The results of sonography were compared with those of laparoscopy.

## 3. Results

We identified a total of 22 patients with 26 impalpable testes, including 4 patients with bilateral impalpable testes, who underwent ultrasonography during the study period. Average patient age at the time of laparoscopy was 31 months. Ultrasonography was successful in localizing 8 of 26 (31%) testes (Table 1).

Only 6 of 26 (23%) testes explored by laparoscopy were identified by sonography, with five of the detected testes localized at the inguinal ring on laparoscopy. In contrast, 7 of the 18 (38.9%) testes not seen on ultrasonography were at the internal ring, whereas two testes identified by ultrasonography were absent during surgical exploration.

## 4. Discussion

Cryptorchidism is one of the most common genitourinary disorders in young boys. Although the management of boys with palpable testes has been standardized, there are no formal guidelines for the management of boys with nonpalpable testes [13].

Orchiopexy is necessary, whether or not ultrasonography localizes a testis. If ultrasonography fails to demonstrate a viable testis, surgical exploration is still necessary, because neither CT nor MRI has been shown to be reliable in diagnosing a vanishing testis. Consequently, routine preoperative imaging is generally unnecessary, although obese boys may require imaging, since physical examination for an undescended testis can be difficult [14]. Nevertheless, a physical examination, even for obese children, is the most important aspect of presurgical assessment of a boy with an undescended testis [15].

Laparoscopy is currently the most reliable diagnostic modality used in the management of impalpable testes. This method clearly shows the anatomy and provides visual information upon which a definitive decision can be based [16].

It was found that sonography is unnecessary in boys with a nonpalpable testis, because it rarely, if ever, localizes a true nonpalpable testis, and it does not alter the surgical approach in these patients [14]. Ultrasound does not reliably localize a nonpalpable testis and does not rule out an intra-abdominal testis. Eliminating the use of ultrasound will not change the management of boys with nonpalpable cryptorchidism, but it will decrease health care expenditures [17]. In our hands, ultrasonography identified only 6 of 26 (23%) testes, a percentage lower than observed previously [14, 18].

Brody [19] proposed a “top-5 list” in which he called for all medical specialties to identify and recommend against heavily used and expensive diagnostic tests that offer little benefit to patients. We believe that the use of ultrasound to evaluate cryptorchidism meets these criteria [15].

## Figures and Tables

**Table 1:** Comparison of ultrasonographic and operative findings.

		Operative findings				
Ultrasound findings		Absent testis	High	Low	At internal ring	Total
Not visualized		9	1	1	7	18
Deep to inguinal ring		—	—	—	2	2
In inguinal canal		1	—	2	2	5
At external ring		1	—	—	—	1

Total		11	1	3	11	26
